# Profiling an Enhanced IL‐6 Expression Distinguishes Host Susceptibility to Primary and Secondary Infections of the Dengue Virus in an Ex Vivo Whole‐Blood Coculture Model

**DOI:** 10.1155/jotm/9350179

**Published:** 2026-01-02

**Authors:** Josephine Diony Nanda, Ming-Kai Jhan, Rahmat Dani Satria, Yung-Ting Wang, Tzong-Shiann Ho, Herdiantri Sufriyana, Emily Chia-Yu Su, Chiou-Feng Lin

**Affiliations:** ^1^ Department of Microbiology and Immunology, School of Medicine, College of Medicine, Taipei Medical University, Taipei 110, Taiwan, tmu.edu.tw; ^2^ Department of Parasitology, Faculty of Medicine, Public Health and Nursing, Universitas Gadjah Mada, Yogyakarta 55281, Indonesia, ugm.ac.id; ^3^ Department of Clinical Pathology and Laboratory Medicine, Faculty of Medicine, Public Health and Nursing, Universitas Gadjah Mada, Yogyakarta 55281, Indonesia, ugm.ac.id; ^4^ Clinical Laboratory Installation, Dr. Sardjito Central General Hospital, Yogyakarta 55281, Indonesia, ugm.ac.id; ^5^ Department of Paediatrics, National Cheng Kung University Hospital, College of Medicine, National Cheng Kung University, Tainan 704, Taiwan, ncku.edu.tw; ^6^ Department of Paediatrics, National Cheng Kung University Hospital Dou-Liou Branch, College of Medicine, National Cheng Kung University, Yunlin 640, Taiwan, ncku.edu.tw; ^7^ Center of Infectious Disease and Signalling Research, National Cheng Kung University, Tainan, Taiwan, ncku.edu.tw; ^8^ Institute of Biomedical Informatics, College of Medicine, National Yang Ming Chiao Tung University, Taipei, Taiwan, medcol.mw; ^9^ Graduate Institute of Biomedical Informatics, College of Medical Science and Technology, Taipei Medical University, Taipei, Taiwan, tmu.edu.tw; ^10^ Clinical Big Data Research Center, Taipei Medical University Hospital, Taipei, Taiwan, tmuh.org.tw; ^11^ Graduate Institute of Medical Sciences, College of Medicine, Taipei Medical University, Taipei 110, Taiwan, tmu.edu.tw; ^12^ Core Laboratory of Immune Monitoring, Office of Research & Development, Taipei Medical University, Taipei 110, Taiwan, tmu.edu.tw

**Keywords:** DENV, ex vivo, IL-6, infection, NS1

## Abstract

Dengue virus (DENV) infection can potentially lead to severe dengue disease due to the risk of antibody‐dependent enhancement. This study reports a comparative analysis of the host cytokine/chemokine response triggered by primary and secondary DENV infections using an artificial ex vivo whole‐blood coculture model to simulate viremia during the acute febrile phase of infection. Using ex vivo primary and secondary DENV infection modes, a dengue‐specific customized multiplex cytokine/chemokine assay was employed. Secondary infection did not exacerbate DENV‐induced hematological and cytopathological changes, such as alterations in complete blood count, intracellular vacuolization, and thrombophagocytosis. However, cytokine/chemokine assay revealed a significant increase in the production of MIP‐1*α*, MIP‐1*β*, IL‐6, TNF‐*α*, and RANTES. Notably, a substantial decrease in NS1 levels indicated the neutralization effect in individuals with prior DENV exposure or secondary infection group, especially in some cases of secondary infection. This was accompanied by pre‐existing anti‐E antibodies, highly associated with IL‐6 overproduction. These findings support the potential strategy of assessing DENV susceptibility using NS1 and IL‐6 using an ex vivo method.

## 1. Introduction

The dengue virus (DENV), transmitted by infected *Aedes*​ mosquitoes in tropical and subtropical regions, infects people each year and poses a global threat of vector‐borne disease in dengue risk [1]. In 2023, an international total of 6.43 million dengue cases and 6892 deaths were reported, averaging 56,672 cases and 28.45 deaths per million people [2]. The complexity of DENV pathogenesis and disease development has so far limited antiviral treatments, with only two vaccines currently approved [3, 4]. The first vaccine, Dengvaxia (CYD‐TDV), is conditionally administered to people aged 9–45 who have laboratory‐confirmed reinfections and live in dengue‐endemic areas [5]. However, the vaccine’s effectiveness is mixed, as it has been associated with increased severity in individuals who had not previously been infected [6, 7].

A primary factor complicating dengue treatment and vaccination is an antibody‐dependent enhancement (ADE), a pathogenic mechanism that can worsen DENV infection, particularly in severe cases [8]. This phenomenon is commonly observed in secondary infection, where subneutralizing antibodies (sub‐nAbs) facilitate virus entry to immune cells, enhancing replication. Although a recent report in pediatric patients showed a balanced severity incidence between primary and secondary infections [9], this trend does not extend to adults and the elderly. Given the antigenic diversity of DENV serotypes, secondary infection in individuals with nonneutralizing antibodies (non‐nAbs) or antibodies at sub‐nAb titers significantly raises the risk of severe dengue via ADE, posing a major challenge to vaccine development and limiting post‐vaccination efficacy [10, 11]. To create a safe dengue vaccine, several criteria must be met: (1) prevention of ADE, (2) broad protection across all DENV serotypes, (3) induction of nAbs at sufficient titers, (4) avoidance of cross‐reactive, non‐nAbs, and (5) minimization of autoimmune risk. These factors are essential to mitigating ADE‐associated complications.

In individuals with prior DENV infection or post vaccination, the status of antiviral humoral immunity remains a critical concern. While such individuals may generate protective neutralizing antibodies (nAbs), they are also prone to producing sub‐nAbs or non‐nAbs, which can facilitate ADE and exacerbate disease severity [12]. Unfortunately, ADE risk cannot be reliably assessed until reinfection occurs, and no straightforward predictive measure currently exists. To address this gap, the study employed an ex vivo whole‐blood coculture model using heparinized venous samples, as previously established [13, 14] to evaluate viral susceptibility. This approach enabled the identification of individuals at risk for secondary infection or those possessing protective immunity, offering a promising tool for preemptive risk stratification in post‐infection and post‐vaccination contexts.

## 2. Materials and Methods

### 2.1. Cells and Virus Culture

Human research was conducted under the Declaration of Helsinki and approved by the Taipei Medical University‐Joint Institutional Review Board (TMU‐JIRB N202003085). Written informed consent was obtained from all study participants (*N* = 12) prior to enrollment, as required by TMU‐JIRB. Blood samples were collected simultaneously using sodium heparin vacutainer tubes (Becton Drive Vacutainer, Franklin Lakes, NJ, USA). None of the subjects enrolled in this study had received dengue vaccination prior to sample collection. BHK21 [C‐13] cells (ATCC CCL‐10) were maintained in Dulbecco’s Modified Eagle Medium (DMEM, Thermo Fisher Scientific, Waltham, MA, USA) and *Aedes albopictus* clone C6/36 cells (ATCC CRL‐1660) were cultivated in Minimum Essential Medium (MEM, Thermo Fisher Scientific, Waltham, MA, USA) supplemented with 10% fetal bovine serum (FBS, Merck, Sigma‐Aldrich, St. Louis, MO, USA). To maintain DENV serotype 2 (PL046), C6/36 cells were exposed to DENV2 at a multiplicity of infection (MOI) of 0.01. The virus was incubated on C6/36 cell monolayers at 28°C with 5% CO_2_ for 5 days. The concentrated and filtered viral supernatant was stored at −80°C, using Millipore Amicon Ultra centrifugal filters (Merck, Sigma‐Aldrich, St. Louis, MO, USA) for filtration. The titration of purified DENV was conducted in BHK‐21 cells using a plaque assay; the detailed method was described in our previous work [15].

### 2.2. Ex Vivo Whole‐Blood Coculture

An ex vivo model of DENV infection in whole blood (WB) was established, as described previously [13, 14]. Briefly, 400 μL of RPMI medium containing DENV2 (MOI = 1) was added to 200 μL of heparinized WB in a 24‐well plate. A hematology analyzer (XN‐450, Sysmex Corporation, Kobe, Japan) was used to determine total white blood cell (WBC) counts. The samples were placed on a shaker and incubated for 24 h at 37°C. Culture supernatants were then collected to measure cytokine production.

### 2.3. Antibody Detection

The measurement of anti‐NS1 (R&D Systems, Minneapolis, MN, USA) and anti‐E (Abcam, Cambridge, UK) antibodies utilizes an indirect human anti‐DENV IgG ELISA technique according to the manufacturer’s instructions. Briefly, the provided 96‐well plate (comprising 12 strips, each with eight wells) is precoated with DENV2 NS1 or E antigens. After the serum samples were added, a biotinylated secondary antihuman IgG antibody was introduced. This secondary antibody binds to any human IgG antibodies attached to the antigen‐coated plate, forming a biotinylated complex. A TMB (3,3′,5,5′‐tetramethylbenzidine, R&D Systems) substrate was then added. This produces a blue color, the intensity of which corresponds to the quantity of antibodies in the sample.

### 2.4. CBC Examination

To assess changes in cell expression in WB, gently mixed WB cultures were analyzed 24 h post inoculation. Complete blood count (CBC) testing was performed using the XN‐450 hematology analyzer (Sysmex Corporation, Kobe, Japan) according to the manufacturer’s instructions. Parameters included the proportions of various WBC components—lymphocytes, monocytes, neutrophils, eosinophils, and basophils—as well as platelet count.

### 2.5. Hematological Examination

After 24 h of ex vivo DENV infection, a 3 mm drop of WB from each sample was placed at one end of a slide and spread across its width. Following air‐drying, all smears were stained with Wright‐Giemsa stain (Tonyar Biotech, Taipei, Taiwan). Cells were then photographed and counted using an Olympus CX23 microscope (Olympus, Tokyo, Japan).

### 2.6. Multiple Cytokine/Chemokine Measurement

Frozen samples were thawed prior to use, and cytokine and chemokine levels were measured using a custom multiplex human cytokine/chemokine magnetic bead panel (Merck Millipore, Billerica, MA, USA) as previously described [16]. The panel measured IFN‐*γ*, IL‐10, IL‐6, IL‐8, TNF‐*α*, CXCL10/IP10, IL‐4, CCL2/MCP‐1, CCL4/MIP‐1*β*, CCL5/RANTES, IFN‐*α*2, GM‐CSF, VEGF, IL‐15, IL‐13, IL‐1R*α*, IL‐18, IL‐12, CXCL9/MIG, CCL3/MIP‐1*α*, IL‐17, IL‐7, IL‐1*β*, and IL‐2. Raw data were obtained as relative fluorescence intensities and converted to cytokine concentrations (pg/mL) using a standard curve based on reference concentrations. Concentrations below the detection limit were set to 0 pg/mL.

### 2.7. Viral NS1 Detection

Viral NS1 levels were measured using a DENV NS1 ELISA kit (Arigo biolaboratories, Hsinchu, Taiwan) according to the manufacturer’s instructions. Quantification was finalized by adding 100 μL of 3,3′,5,5′‐tetramethylbenzidine substrate as a chromogenic indicator.

### 2.8. Statistical Analysis

Groups were compared using Student’s paired *T*‐tests, and the results are presented as mean ± standard deviation (SD). Statistical significance was defined with a threshold *p* value of 0.05. To further understand the relationship between various immunological markers, principal component analysis (PCA) was employed. In this assay, cytokine levels were normalized to capture dominant variation, with PC1 used to interpret cytokine contributions. Hierarchical clustering was applied to explore sample similarities. Associations between PC1 scores and antibody levels were assessed by linear regression. Cytokines were grouped by function, and PC1 loadings were visualized across groups. Analyses were conducted using Prism 8 software from GraphPad and R version 4.4.1.

## 3. Results

### 3.1. Identification of Cell Responses to Primary and Secondary Infections of DENV Using an Ex Vivo Whole‐Blood Coculture Model

To assess susceptibility and host responses to potential DENV infection in individuals, this study utilized a previously established ex vivo whole‐blood coculture model [13, 14]. After a 24‐h incubation with DENV2 (MOI = 1), we examined relevant viral markers, hematological changes, and cytokine/chemokine production associated with DENV infection [16] (Figure 1(a)). We recruited two groups of participants: those previously infected with DENV or secondary infection (*n* = 7) and the other with no history of DENV infection or primary infection (*n* = 5). First, we used a sandwich ELISA to measure anti‐NS1 antibodies in serum to confirm the groups’ distinction. The previously infected group showed significantly higher antibody titers, while the control group was negative (Figure 1(b)), supporting our model’s basis for examining primary (seronegative) and secondary (seropositive) infection responses.

Figure 1Secondary infection does not promote immune cell abnormalities in acute phase of dengue infection. (a) Research design and experimental methodology: ex vivo blood coculture infection model. (b) Detection of pre‐existing DENV anti‐NS1 antibodies in subjects’ serum (*n* = 12): negative indicates primary infection (*n* = 5), while positive indicates secondary infection (*n* = 7) used in this study (*p* < 0.001). (c), (d) Effects of viral infection on peripheral blood cell count and morphology with intracellular vacuolization and thrombophagocytosis: differences between primary (*n* = 3) and secondary (*n* = 3) infections. Ns, not significant.

**Figure:**
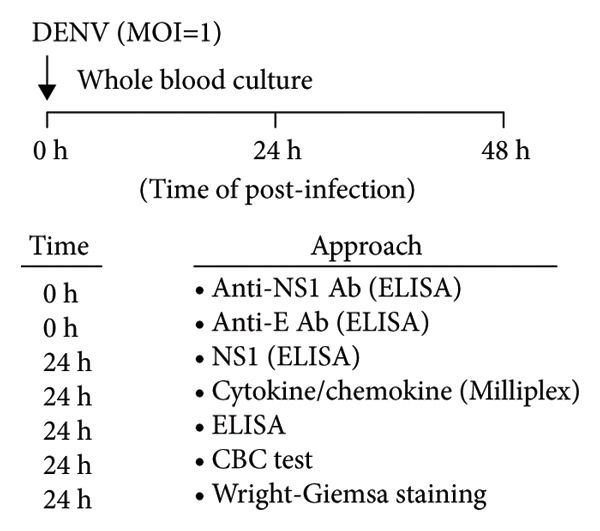
(a)

**Figure:**
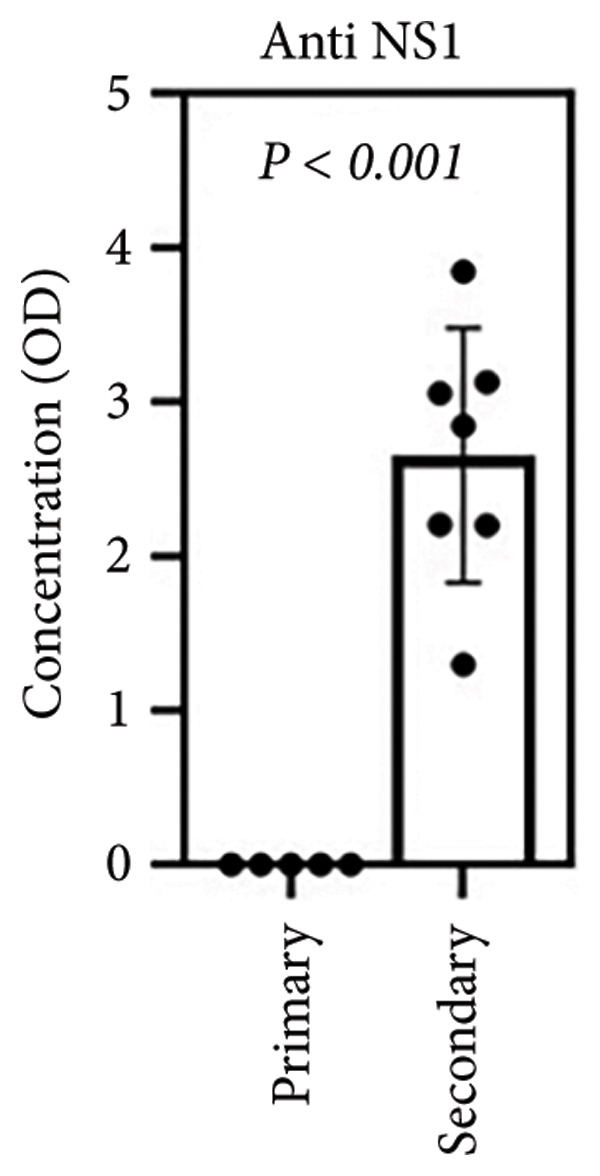
(b)

**Figure:**
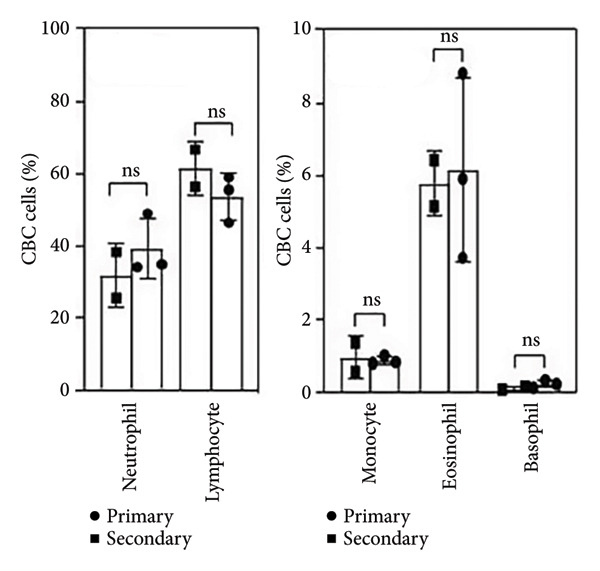
(c)

**Figure:**
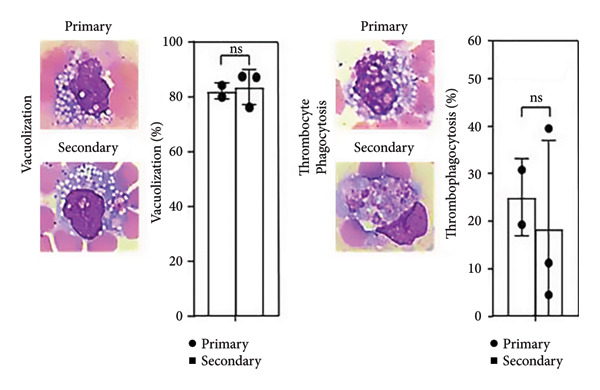
(d)

Our previous research indicated a reduction in neutrophils in an ex vivo whole‐blood coculture model following DENV infection [13, 14]. Nevertheless, CBC results here showed no significant changes in major blood cell populations between primary and secondary infections (Figure 1(c)). Despite observing vacuolization and thrombophagocytosis in infected blood cells in our model [13, 14], no pathological differences were detected between primary and secondary infections within 24 h (Figure 1(d)). The findings suggest that early‐stage hematological pathology may not distinguish primary and secondary infections.

### 3.2. Characterization of Immune Responses to Primary and Secondary Infections Using the Ex Vivo Coculture Model

Using the same infection model, we collected the culture supernatant (containing plasma) and assessed cytokine/chemokine levels with our lab’s custom dengue‐related cytokine/chemokine detection panel [16]. This panel was designed based on clinical findings of significant cytokine/chemokine presence in dengue patients’ blood. Our results revealed elevated levels of multiple cytokines/chemokines across all dengue‐infected samples (*n* = 12) (Table 1); however, distinct cytokine/chemokine expression profiles were evident between primary and secondary infection groups (Figure 2(a)). Compared to primary infections, secondary infections exhibited significantly higher levels of five cytokines/chemokines: MIP‐1*α*, MIP‐1*β*, TNF‐*α*, IL‐6, and RANTES (Figure 2(b)), highlighting that cytokine expression profiles can differentiate primary and secondary infections in early stages (within 24 h).

**Table 1:** Expression of cytokine/chemokine in an ex vivo blood coculture infection model.

Cytokine	Mock (*n* = 12, pg/mL)	DENV (*n* = 12, pg/mL)	Primary (*n* = 5, pg/mL)	Secondary (*n* = 7, pg/mL)	*p* value mock versus DENV	*p* value primary versus secondary
GM‐CSF (CSF‐2)	45.88	80.24	90.73	71.49	0.235	0.537
IFN‐*α*	4.02	6.00	7.84	4.47	0.608	0.931
IFN‐*γ*	7.69	87.88	73.21	100.11	0.004^∗∗∗^	0.537
IL‐1*β*	0.37	8.90	5.07	12.08	*p* < 0.001^∗∗∗^	0.429
IL‐1R*α*	1.34	21.12	17.06	24.50	*p* < 0.001^∗∗∗^	0.792
IL‐2	1.15	9.70	8.22	10.76	0.002^∗∗∗^	0.432
IL‐4	0.49	1.06	1.40	0.82	0.178	0.755
IL‐6	2.05	1363.57	471.51	2255.64	*p* < 0.001^∗∗∗^	0.032^∗^
IL‐7	0.87	0.84	1.27	0.65	0.853	0.667
IL‐8 (CXCL‐8)	538.33	10,193.33	9693.80	10,550.14	*p* < 0.001^∗∗∗^	1.000
IL‐10	6.17	26.62	18.63	34.61	0.019^∗^	0.548
IL‐12	1.31	1.24	1.22	1.25	0.740	0.530
IL‐13	3.63	6.11	4.58	7.38	0.091	0.177
IL‐15	1.44	1.62	1.38	1.79	0.713	0.639
IL‐17	0.54	1.48	1.28	1.68	0.014^∗^	0.548
IL‐18	1.15	0.13	0.13	0.13	0.422	0.730
IP‐10 (CXCL‐10)	51.81	538.98	833.85	328.36	0.001^∗∗∗^	0.755
MCP‐1 (CCL‐2)	7590.67	21,727.50	18,439.60	25,015.40	0.009^∗∗^	0.690
MIG (CXCL‐9)	333.37	1444.08	1584.86	1343.52	*p* < 0.001^∗∗∗^	1.000
MIP‐1α (CCL‐3)	22.59	2287.91	494.80	3782.17	*p* < 0.001^∗∗∗^	0.004^∗∗^
MIP‐1*β* (CCL‐4)	31.12	4724.99	1804.97	6810.71	*p* < 0.001^∗∗∗^	0.010^∗∗^
RANTES (CCL‐5)	3186.42	5178.25	3518.20	6364.00	0.028^∗^	0.048^∗^
TNF‐*α*	5.64	319.75	153.17	486.32	*p* < 0.001^∗∗∗^	0.008^∗∗^
VEGF (VEGFA)	57.41	122.04	115.03	127.04	0.060	0.755

^∗^ *p* < 0.05; ^∗∗^ *p* < 0.01; and ^∗∗∗^ *p* < 0.001.

Figure 2Comparative cytokine profile in healthy individuals and dengue patients, with primary versus secondary infection. (a) Cytokine expression in ex vivo whole‐blood coculture infection model: infection status and comparison between primary (*n* = 5) and secondary (*n* = 7) infections. (b) Significant differences in selected cytokine expression: based on infection status (Mock *n* = 12, DENV *n* = 12) and primary (*n* = 5)/secondary (*n* = 7, red) infection. ^∗^ *p* < 0.05; ^∗∗^ *p* < 0.01; and ^∗∗∗^ *p* < 0.001.

**Figure:**
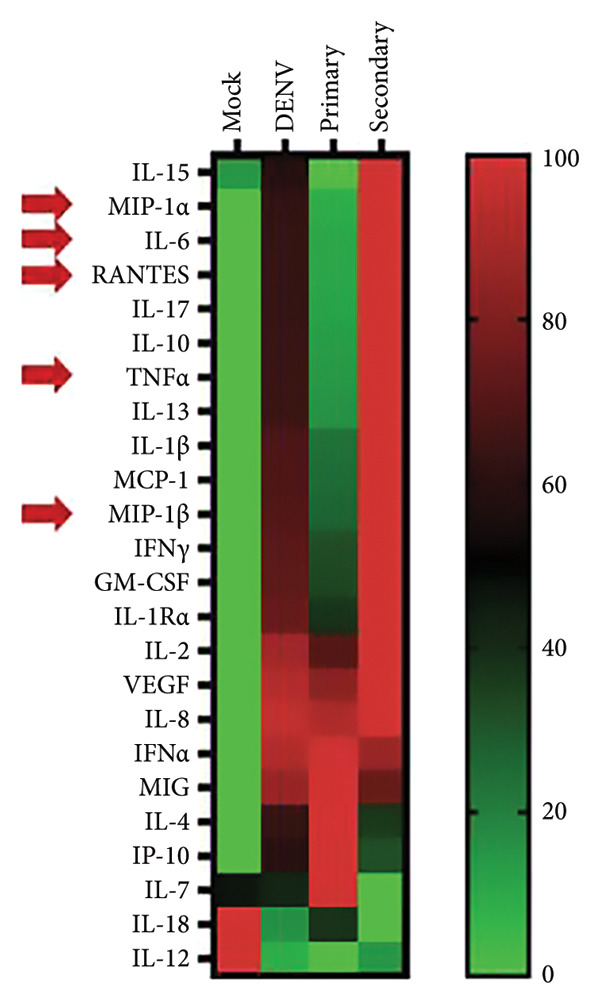
(a)

**Figure:**
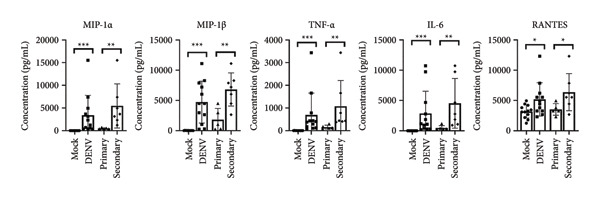
(b)

### 3.3. Association of Cell Sensitivity and Induced Immune Responses With Primary and Secondary Infections

Based on the observed cytokine/chemokine expression differences, we hypothesized that there are different cell susceptibility patterns between primary and secondary infections. Next, we evaluated secreted NS1 protein expression, which was defined as a marker for viral replication and dengue disease severity [17], in the culture medium by sandwich ELISA to confirm group differences. However, the secondary infection group showed a trend of decreased NS1 levels, while the primary infection group showed higher levels (Figure 3(a)). As secondary infection was established in this study, no further ADE of DENV infection was observed. Notably, a negative correlation was observed between baseline anti‐E antibody levels and NS1 protein levels (*r = *−*0.82, p = 0.024*) in infected samples (Figure 3(b)). This result suggests that pre‐existing anti‐E antibody levels or NS1 protein expression could distinguish host‐susceptible responses between primary and secondary infections in the early stage (within 24 h).

Figure 3Expression of DENV NS1 and its correlation with the pre‐existing viral antibodies. (a) There was no significant difference in NS1 levels between primary and secondary infection modeling (*p* = 0.135). (b) Significant correlation between NS1 and pre‐existing anti‐E antibody concentration was observed (*n* = 7) (*p* = 0.024, *r* = −0.82). Statistical significance set at *p* < 0.05.

**Figure:**
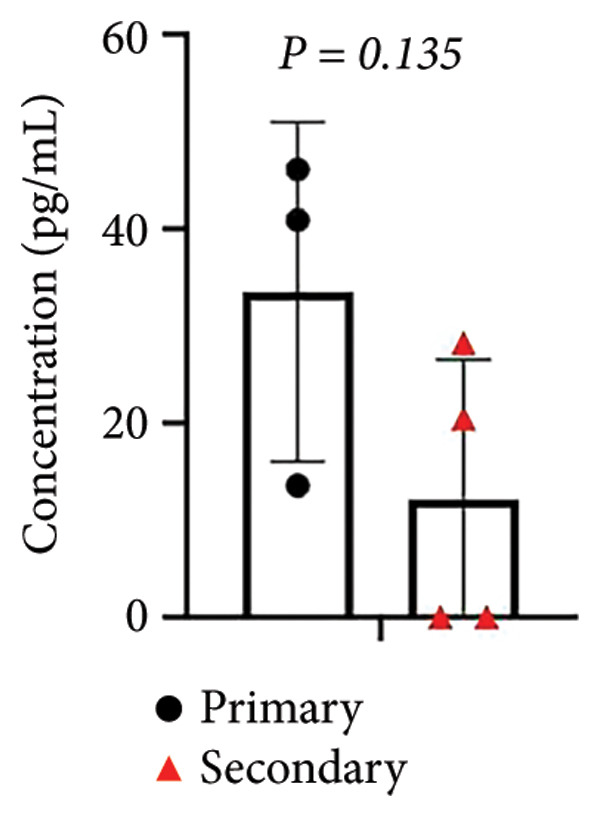
(a)

**Figure:**
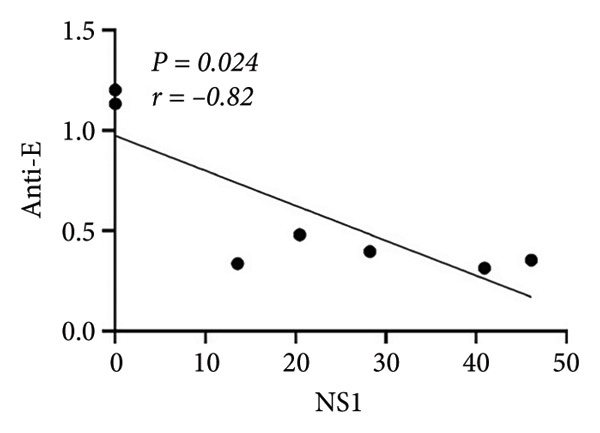
(b)

With a detailed understanding of host immune responses during early‐stage primary and secondary infections (within 24 h), we analyzed correlations between participants’ anti‐E antibody titers, induced cytokine/chemokine levels, and viral factors (Figure 4(a)). A key finding was the positive correlation (*r = 0.975, p* < 0.001) between IL‐6 expression and anti‐E antibody titers (Figure 4(b)), along with a negative correlation (*r = *−*0.82, p* = 0.023) between IL‐6 and NS1 protein production (Figure 4(c)). The results imply that IL‐6 expression not only differentiates between early primary and secondary infections but also has potential associations with pre‐existing antiviral immunity.

Figure 4Correlations between the pre‐existing anti‐E antibodies and inducible host/viral factors. (a) The heatmap shows the correlation coefficient between pre‐existing anti‐E antibodies, inducible DENV NS1, and several cytokines obtained from an ex vivo whole‐blood coculture infection model. (b) Significant positive correlation was found between pre‐existing anti‐E and IL‐6 concentrations; meanwhile, (c) DENV NS1 correlates negatively with IL‐6. Statistical significance set at *p* < 0.05.

**Figure:**
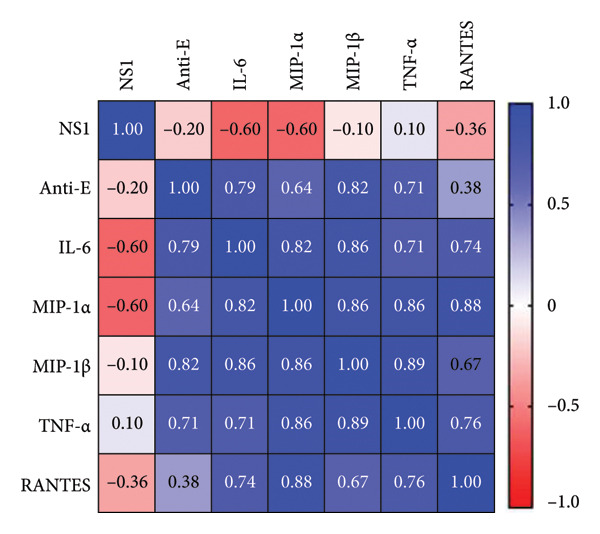
(a)

**Figure:**
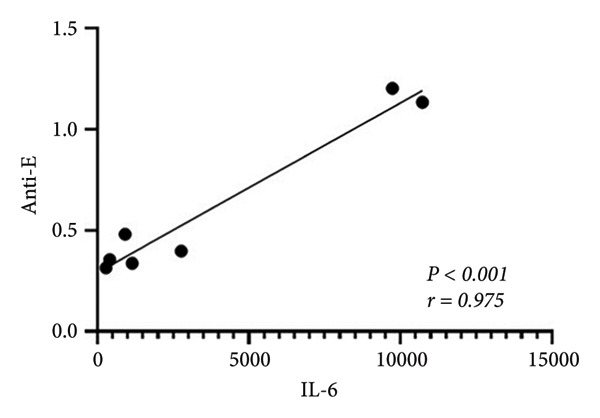
(b)

**Figure:**
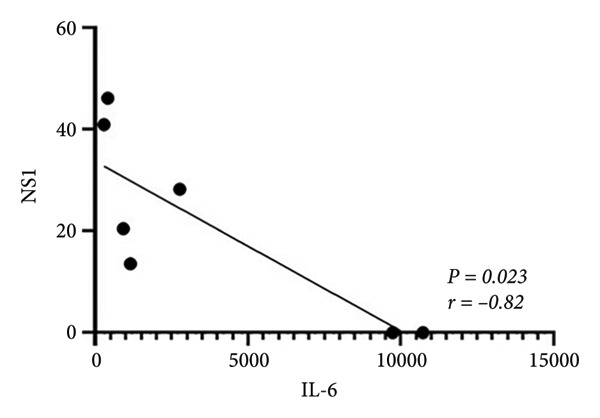
(c)

To further investigate variation in cytokine expression patterns, PCA was performed on normalized cytokine levels. The PCA biplot revealed clear separation between DENV‐infected and mock samples along PC1, which accounted for 40% of the variance (Figure 5(a)). PC1 and PC2 scores also showed distinct distributions between conditions (Figures 5(b), 5(c)), and hierarchical clustering based on cytokine profiles demonstrated consistent group‐level differences (Figure 5(d)). PC1 scores were positively correlated with anti‐E antibody levels among DENV‐infected samples (Figure 5(e), Table 2). In either primary or secondary infection, PC1 scores were significantly higher in DENV‐infected samples compared to mock (Figure 5(f), Table 2). The correlation between PC1 and anti‐E antibody levels was also distinct between DENV‐infected and mock samples in the secondary infection group (Figure 5(g), Table 2). Cytokines with the greatest contributions to PC1 included IL‐6, IL‐1*β*, and IFNγ, particularly among samples with secondary infection (Figure 5(h)). Several of these cytokines also correlated significantly with IL‐6, indicating an IL‐6–centered immune signature linked to infection status and pre‐existing immunity.

Figure 5Cytokine profiles distinguish DENV infection from mock condition. (a) PCA biplot shows separation of DENV and mock conditions, annotated by specimen ID. (b), (c) Density plots of PC1 and PC2 scores, respectively, stratified by condition. (d) Hierarchical clustering of all samples based on cytokine profiles, colored by condition. (e) PC1 score is positively associated with anti‐E O.D. in DENV but not mock samples. (f) PC1 scores are significantly higher in DENV‐infected samples compared to mock, but not significantly different between primary and secondary infections. (g) In DENV‐infected samples, PC1 scores correlate positively with anti‐E antibody levels only among secondary infections, while PC1 scores correlate positively with anti‐E antibody levels among both primary and secondary infections. (h) Loadings of cytokines on PC1 and PC2 indicate IL‐6, IL‐1B, and IFNγ as top contributors to DENV‐driven variation among secondary infections. ^∗^Cytokines that were significantly correlated to IL‐6; O.D., optical density; PCA, principal component analysis; and DENV, dengue virus.

**Figure:**
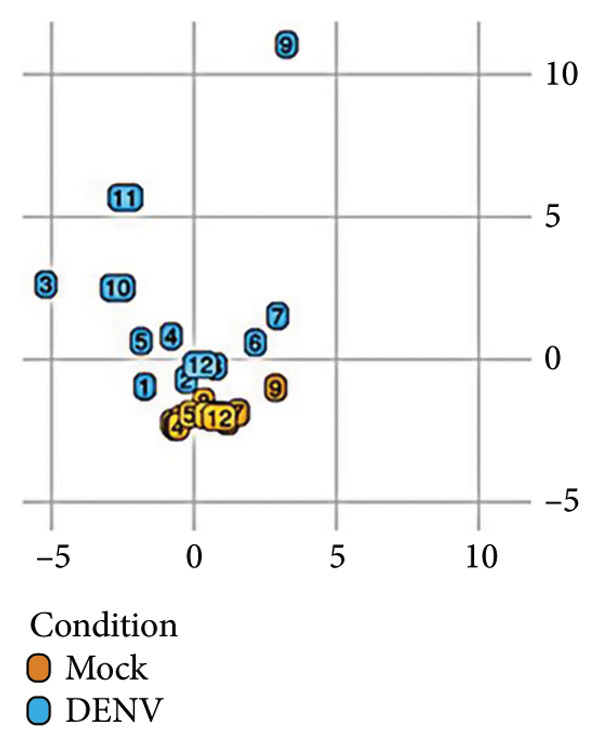
(a)

**Figure:**
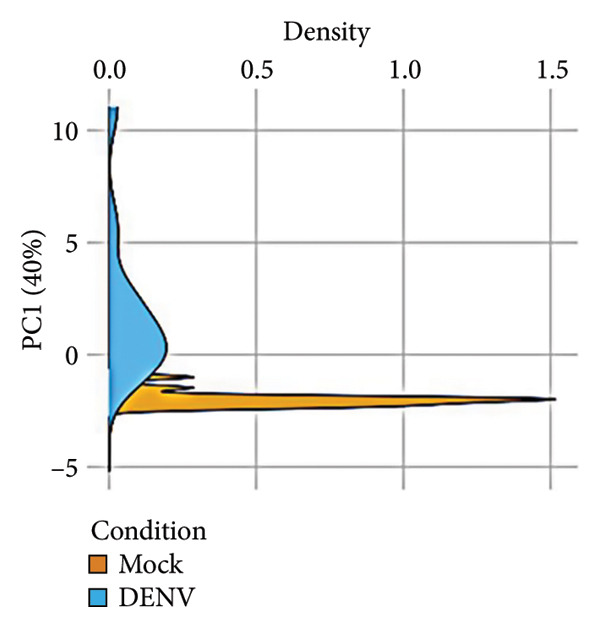
(b)

**Figure:**
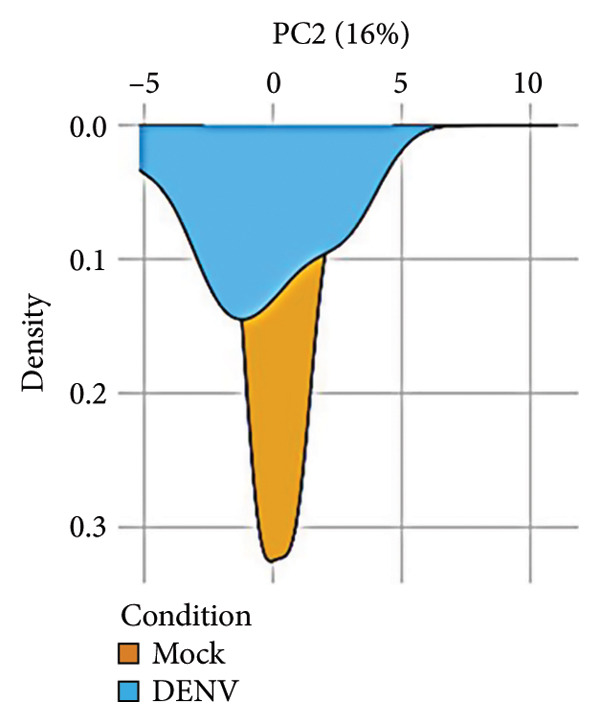
(c)

**Figure:**
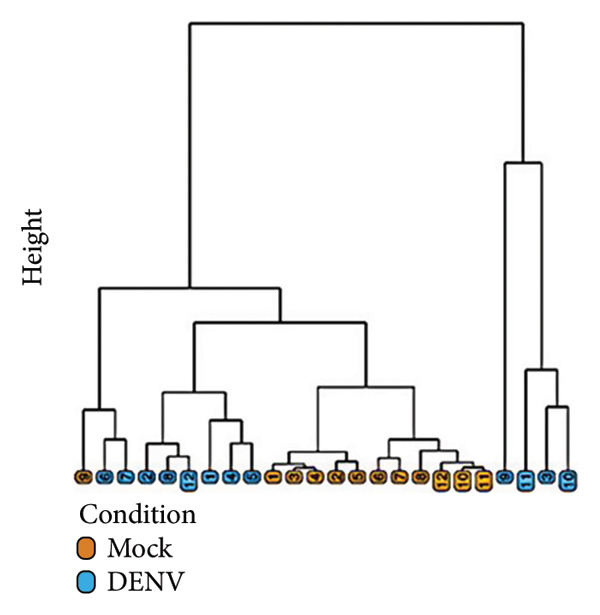
(d)

**Figure:**
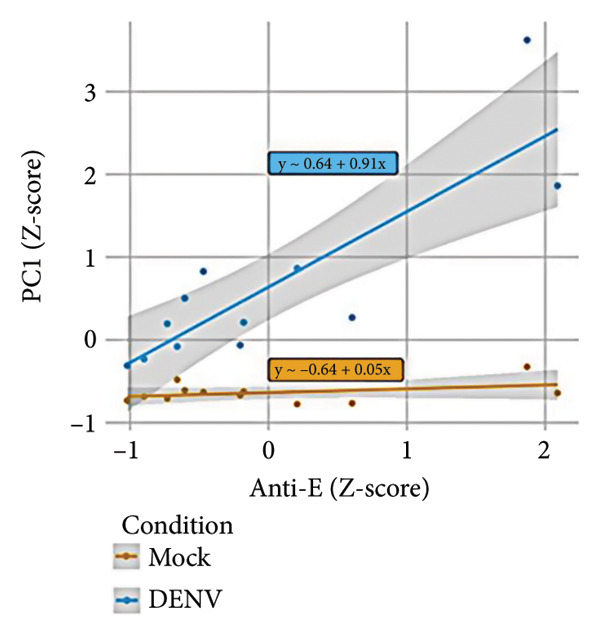
(e)

**Figure:**
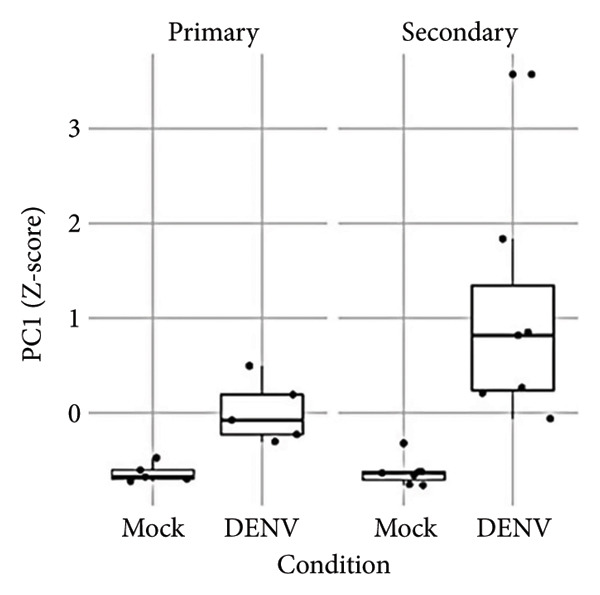
(f)

**Figure:**
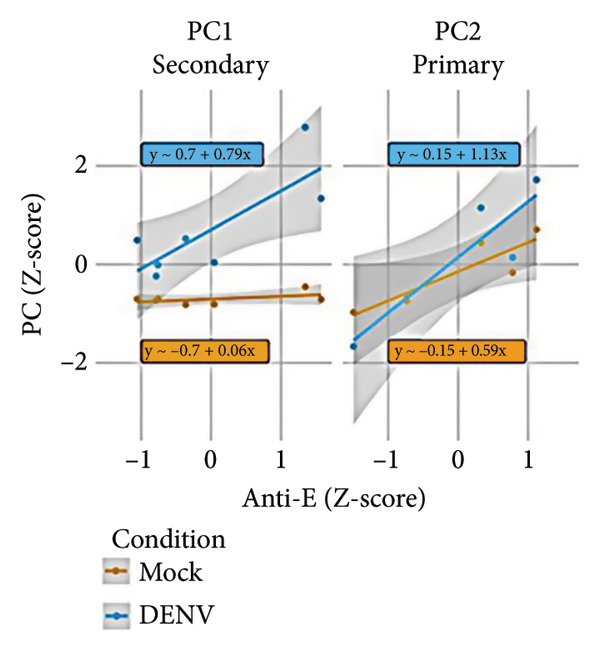
(g)

**Figure:**
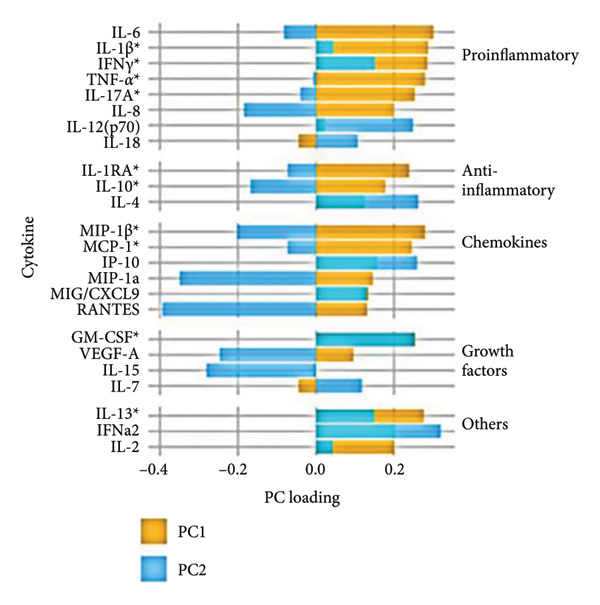
(h)

**Table 2:** Linear regression between PC1 scores and antibody levels.

Variable	Strata	Estimate†	*p* value
Anti‐E	Mock	0.05 (95% CI −0.02, 0.12)	0.225
	DENV	0.91 (95% CI 0.56, 1.27)	< 0.001^∗^
Anti‐NS1	Mock	−0.01 (95% CI −0.08, 0.07)	0.837
	DENV	0.41 (95% CI −0.21, 1.03)	0.221
NS1	Mock	−0.04 (95% CI −0.11, 0.03)	0.266
	DENV	−0.07 (95% CI −0.78, 0.64)	0.851
Condition	Primary	0.65 (95% CI 0.35, 0.95)	0.003^∗^
	Secondary	1.7 (95% CI 0.75, 2.64)	0.004^∗^
Anti‐E	Mock‐primary	0.16 (95% CI −0.03, 0.35)	0.192
	DENV‐primary	0.63 (95% CI 0.14, 1.11)	0.085
	Mock‐secondary	0.06 (95% CI −0.03, 0.14)	0.259
	DENV‐secondary	0.79 (95% CI 0.27, 1.32)	0.031^∗^

^∗^ *p* value < 0.05; †, Z‐score.

## 4. Discussion

The ongoing burden of severe dengue presents urgent medical challenges, particularly in detection strategies that can predict disease severity in those who might be infected. Therapeutically, the lack of effective antiviral drugs remains the biggest challenge to mitigate the progression of severe dengue disease [3]. From a preventive standpoint, while eradicating sources of infection might be essential, long‐term safety evaluations of vaccine candidates are still challenging, considering the risks associated with secondary infections and enhancement effects following the vaccination [4, 6]. This study used ex vivo whole‐blood coculture model to simulate primary and secondary DENV infections, enabling an assessment of host response sensitivity to infection. Beyond the influence of pre‐existing anti‐E antibodies, our findings indicate that the anti‐NS1 produced during infection and the production of host cytokine IL‐6 are associated with the stimulation of antiviral immune response. Prospectively, individualized testing of blood cell susceptibility to artificially induced DENV infection in the lab may offer a novel approach to estimating potential disease severity.

The antiviral immune response elicited by secondary dengue infections or post vaccination may provide adequate protection against subsequent exposures; however, it also carries the potential risk of ADE. At present, no validated detection method exists to assess this risk in clinical settings. Our study demonstrated that dengue infection elicited a higher anti‐E antibody response compared to the control group, particularly in the secondary infection. Moreover, elevated levels of IL‐6, I‐1*β*, and IFN‐*γ* were observed alongside increased anti‐E antibody production. This implies that in the secondary infection, the increased cytokine production might be attributed to an attempt by the immune system to control viral activity, although this antibody may instead facilitate viral entry to immune cells via Fcγ receptor, raising concerns about ADE.

Our earlier study using an ex vivo infection model demonstrated that DENV induces reduced neutrophil expression, accompanied by distinct cellular changes, such as vacuolization and thrombophagocytosis [13, 14]. Although secondary infection trials have shown activation of antiviral immune responses, this does not appear to prevent early hematological abnormalities. This suggests that hematologic cellular alteration may arise not from viral replication during the later stages of infection but rather from initial interactions between the virus and blood cells that trigger pathological responses. Notably, our prior findings implicated the cytokine TNF‐α as a key mediator of these cellular effects, with both primary and secondary infections influencing its production. Nevertheless, the molecular mechanisms regulating DENV–blood cell interactions remain unclear and warrant further investigation.

In secondary dengue infection, the host immune response is known to be more rapid and robust than that observed in primary infection, primarily due to the presence of pre‐existing immunological memory cells. B and T lymphocytes exhibit elevated activation levels and accelerated response dynamics, although the memory responses involved are from different viral serotypes [18]. This cellular activation leads to an earlier and amplified production of proinflammatory cytokines, such as IL‐6, TNF‐*α*, and IFN‐*γ*, which are mobilized to suppress viral infection. Studies have shown that in secondary infections, these cytokines begin to peak as early as day three post symptom onset, coinciding with the transition into the critical phase. This peak is followed by a decline that converges to a comparable level across all degrees of dengue severity by the sixth day post onset [19]. The inability to downregulate innate cytokines, particularly TNF‐*α*, IL‐6, and IP‐10, during the late phase of illness has been associated with delayed resolution of serum inflammatory mediators in patients with severe dengue [20].

Early immune response is a critical determinant of disease progression. Elevated levels of IL‐10 during the initial phase of the infection have been correlated with increased disease severity [21, 22], as its potent anti‐inflammatory properties interfere with the host antiviral response. On the other hand, IL‐6, as a potent inflammatory cytokine, is widely reported as a biomarker for dengue severity [21–23]. Notably, IL‐6 levels are reported to be significantly higher in secondary infections, particularly those involving DENV2, but not DENV1 [24]. Although IL‐6 is reported not to be directly linked with vascular leakage [25], some studies associate its increase with bleeding tendencies [23, 26]. Another report, however, presents that the elevation of IL‐6 has been detected in 65% of patients presenting with ascites or pleural effusion [27]. Something to be aware of is that the majority of data on IL‐6 kinetics are derived from survivors, which may obscure key regulatory failures in fatal cases. In our view, what governs clinical outcome is not merely the magnitude of IL‐6 elevation, but the host’s ability to enact an appropriate ‘braking’ response‐reducing IL‐6 levels once the initial infection has been addressed. This immunological regulation may represent a pivotal threshold between recovery and escalation into severity.

In endemic regions, profiling dengue antibody responses offers critical insight into the dynamics of host immunity and potential vulnerability to sequential infections. While the anti‐NS1 antibody response is commonly used to detect prior dengue infection, its expression may relate to potential protective or pathogenic properties. For instance, anti‐NS1 may neutralize NS1‐mediated cellular inflammation or activate the complement pathway against viral infection by targeting the NS1 protein [28, 29]. Conversely, anti‐NS1 antibodies could trigger autoimmune effects, contributing to coagulation abnormalities or hemorrhagic manifestations [30]. Another critical antibody response is the dual role of anti‐E antibodies, focusing generally on neutralizing antibody effects and ADE pathology for non‐nAb [8]. For individuals previously infected or vaccinated against dengue, anti‐E antibody responses are anticipated as a humoral immune outcome. Concerns remain about the possibility of protection or enhanced infection in response to unexpected secondary infections. Consequently, establishing a standard lab‐based test would hold potential application value in clinical settings. While the findings offer valuable insights, they should be interpreted in light of several limitations. First, the study was constrained by a limited sample size and the absence of DENV serotype identification, which may affect the generalizability of the results. Future studies should incorporate a larger cohort and include serotype characterization to better account for the complexity of cross‐serotype secondary infections. Additionally, further validation study is needed to assess the utility of this ex vivo model in evaluating susceptibility and protective immunity following primary (anti‐NS1 seronegative) or secondary (anti‐NS1 seropositive) infections.

## 5. Conclusion

This study provided the first experimental model for evaluating host immune responses to primary and secondary dengue infections. Through serological profiling of viral NS1 and host IL‐6, this model enables assessment of individual susceptibility and the potential between protective and pathogenic immune mechanisms.

## Ethics Statement

Human research was conducted under the Declaration of Helsinki and approved by the Taipei Medical University‐Joint Institutional Review Board (TMU‐JIRB N202003085). All participants provided informed consent, as required by TMU‐JIRB.

## Disclosure

All authors read and approved the final manuscript.

## Conflicts of Interest

The authors declare no conflicts of interest.

## Author Contributions

J.D.N., M.K.J., R.D.S., Y.T.W., and T.S.H.: investigation, methodology, conceptualization, and writing–original draft. H.S. and E.C.Y.S.: analysis and methodology. C.F.L.: project administration, supervision, conceptualization, writing–original draft, and editing. Josephine Diony Nanda and Ming‐Kai Jhan contributed equally to this study.

## Funding

This study was supported by grants (NSTC110‐2320‐B‐038‐064‐MY3, 111‐2327‐B‐006‐005, 113‐2320‐B‐038‐027, 113‐2327‐B‐006‐002, and 114‐2320‐B‐038‐060) from the National Science and Technology Council in Taiwan and the Industry‐Academia Cooperation Program (TMU‐MSD A‐111‐112) in the United States.

## Data Availability

The data that support the findings of this study are available from the corresponding author upon reasonable request. The source codes of data analysis in R version 4.4.1 are publicly available (https://github.com/herdiantrisufriyana/denv_il6).
